# Review of the sustainability of food systems and transition using the Internet of Food

**DOI:** 10.1038/s41538-018-0027-3

**Published:** 2018-10-09

**Authors:** Nicholas M. Holden, Eoin P. White, Matthew. C. Lange, Thomas L. Oldfield

**Affiliations:** 1 UCD School of Biosystems and Food Engineering, University College Dublin, Belfield, Dublin, 4 Ireland; 2 Orbas CTR Ltd, Dublin, Ireland; 3 Department of Food Science and Technology, University of California, Davis, CA 95616 USA

**Keywords:** Environmental impact, Agriculture, Environmental impact, Agriculture

## Abstract

Many current food systems are unsustainable because they cause significant resource depletion and unacceptable environmental impacts. This problem is so severe, it can be argued that the food eaten today is equivalent to a fossil resource. The transition to sustainable food systems will require many changes but of particular importance will be the harnessing of internet technology, in the form of an ‘Internet of Food’, which offers the chance to use global resources more efficiently, to stimulate rural livelihoods, to develop systems for resilience and to facilitate responsible governance by means of computation, communication, education and trade without limits of knowledge and access. A brief analysis of the evidence of resource depletion and environmental impact associated with food production and an outline of the limitations of tools like life cycle assessment, which are used to quantify the impact of food products, indicates that the ability to combine data across the whole system from farm to human will be required in order to design sustainable food systems. Developing an Internet of Food, as a precompetitive platform on which business models can be built, much like the internet as we currently know it, will require agreed vocabularies and ontologies to be able to reason and compute across the vast amounts of data that are becoming available. The ability to compute over large amounts of data will change the way the food system is analysed and understood and will permit a transition to sustainable food systems.

## Introduction

The food we eat today is unsustainable for two reasons: the food system causes unacceptable environmental impacts and it is depleting non-renewable resources. Our food can be regarded as ‘fossil food’ because its production relies on fossil fuel, non-renewable mineral resources, depletion of groundwater reserves and excessive soil loss. The idea of sustainable food systems is at the heart of global efforts to manage and regulate human food supply.^1^ The sustainable development goals focus on a number of critical global issues, but Goal 2 (‘end hunger, achieve food security and improved nutrition and promote sustainable agriculture’), Goal 12 (‘ensure sustainable consumption and production patterns’) and Goal 13 (‘take urgent action to combat climate change and its impacts’) are intimately related to the need to transition global food systems from fossil to sustainable. To understand how to meet the challenge presented by these goals, it is necessary to consider what is meant by ‘sustainable’ in the context of a food system. In 1989, the Food and Agriculture Organisation (FAO) council defined sustainable development as ‘the management and conservation of the natural resource base, and the orientation of technological and institutional change in such a manner as to ensure the attainment and continued satisfaction of human needs for present and future generations. Such sustainable development (in the agriculture, forestry and fisheries sectors) conserves land, water, plant and animal genetic resources, is environmentally non-degrading, technically appropriate, economically viable and socially acceptable’.^2^ The important ideas in this definition are working within the planetary boundary (‘the natural resource base’), having a future-proof system (‘continued satisfaction’, ‘present and future generations’), limiting impacts to those manageable by the buffering capacity of the planet (‘environmentally non-degrading’), considering the financial needs of business stakeholders (‘economically viable’) and compatible with local needs and customs (‘socially acceptable’).

Five principles have been identified to support a common vision for sustainable agriculture and food.^3^ These are: (1) resource efficiency; (2) action to conserve, protect and enhance natural resources; (3) rural livelihood protection and social well-being; (4) enhanced resilience of people, communities and ecosystems; and (5) responsible governance. The aim of this paper is to outline the case for why food systems are not sustainable and to define the case for using technology, specifically internet technologies (hardware and software combined to make the ‘Internet of Food’) to enable the transition of the food system from fossil to sustainable. Increasing population, increasing consumption, a billion malnourished people and agriculture that is concurrently degrading land, water, biodiversity and climate on a global scale^4^ combine to indicate that the fossil food systems we currently rely on are not fit-for-purpose. It is suggested that halting agricultural expansion, closing yield gaps, increasing efficiency, changing diets and reducing waste could lead to a doubling of food production with reduced environmental impacts of agriculture.^4^ To achieve these changes, it is going to be necessary to harness internet technology, in the form of an ‘Internet of Food’, which offers the chance to use global resources more efficiently, to stimulate rural livelihoods, to develop systems for resilience and to facilitate responsible governance by means of computation, communication, education and trade without limits of knowledge and access.

The concept of ‘Internet of Food’ first appeared in peer-reviewed literature in 2011 (based on a search of scopus.com using ‘Internet of Food’ as the search term). It was described by the idea of food items having an ‘IP identify’, which raised the question of how this might influence our eating habits.^5^ Their focus was very much on how the technology could influence food choices and predicted that by 2020 it would be possible to monitor and control food objects remotely through the Internet. It is this technological control of the food system that has real potential to help societal stakeholders (consumers, retailers, processors, producers, shareholders, landowners, indigenous peoples and so on) to engage in the transition of our food system from being fossil to sustainable. The ubiquitous physical tagging and sensing of mass and energy flow in the food system linked to a formal semantic web will allow computation over the whole system to answer questions such as: What was the resource depletion of this product? What is the social impact of eating this product? What food safety procedures have been employed for this product? What and where has wealth been created by the value chain of this product? When these questions can be answered for specific instances of food product types and predicted for new products, then it will be possible to determine whether a specific food system is sustainable or not. The stakeholders demanding answers to these questions are likely to be governance and policy makers and consumers. When these questions can be answered, it will be possible to plan how to manage the evolution of the fundamental life support system (food) from fossil to sustainable in order to support a growing global population.

## Current food systems

To understand the need for a systematic transformation of the food system, it is necessary to detail exactly why it is unsustainable. An overwhelming case can be made for the environmental dimension of the system, but there are also social and economic issues as well. This paper will focus the environmental case (resource depletion and adverse environmental impact that relate to the ‘continued satisfaction’ and ‘environmentally non-degrading’ criteria for sustainable food systems), but similar cases can be made for important social and economic issues as well.

### Resource depletion

The resource depletion case can be made with respect to energy, nutrients, water, soil and land. Each will be summarised in turn. To date, the agri-food system has converted non-renewable fossil fuel energy into food by enabling mechanisation, amplified fertiliser production, improved food processing and safe global transportation.^6^ According to FAO,^7^ the agri-food sector accounts for around 30% of the world’s total energy consumption, with Europe alone accounting for 17% of gross energy consumption in 2013.^8^ Agriculture, including crop cultivation and animal rearing, is the most energy-intense phase of the food system, accounting for nearly one third of the total energy consumed in the food production chain.^9^ To date, renewable energy has had limited penetration of the agri-food sector with fossil fuels accounting for almost 79% of the energy consumed by the food sector.^8^ From an energy perspective, the food system can be regarded as unsustainable (cannot meet the ‘continued satisfaction’ requirement) due to its reliance on fossil energy sources.

By the end of the 20th Century, it was estimated that US-produced ammonia represented 32% of fertiliser nitrogen (N) demand, which was produced by extracting N from the atmosphere as ammonia by a process using hydrogen from natural gas (fossil fuel).^9^ The vast majority of N fertilisers consumed today are still created using fossil fuels and cannot be regarded as sustainable until such times as new technological approaches emerge, which are currently in their infancy.^10,11^ A review of mineral fertiliser reserves concluded that potash reserves (the source of most potassium (K) fertilisers) are of great concern and that it is time to start evaluating other sources of K for agriculture^12^ but others concluded that ‘modern agriculture is currently relying on a non-renewable resource and future phosphate rock is likely to yield lower quality P at a higher price’.^13^ If significant physical and institutional changes are not made to the way we currently use and source P, agricultural yields will be severely compromised in the future. Estimates for when world peak P will be reached range from 2027^14^ to 2033.^13^ Variations in estimations of when peak P will occur are due the constant changing of reserve levels.^12^ The ‘power imbalance’ where just three countries controlling >85% of the known global phosphorus reserves,^15^ a concentration of power far greater than that of crude oil, is also of concern, and it has been concluded that the combined impact increasing demand, dwindling reserves and geopolitical constraints could result in reduced production and supply of chemical P fertilisers and increased global P price.^16^ It is clear that over time horizons of around 50 years the agri-food system is going to face a major nutrient crisis unless reliance of fossil mineral resources is significantly reduced and ultimately eliminated. From a nutrient management point-of-view, the food system can be regarded as unsustainable (cannot meet the ‘continued satisfaction’ requirement) due to its reliance on fossil mineral resources.

Modern food production is reliant on irrigation to a great extent, which according to the UN water programme, accounts for 70% of freshwater withdrawals worldwide.^17^ Excessive removal of groundwater for irrigation is leading to rapid depletion of aquifers in key food-producing regions, such as North-Western India, the North China Plain, Central USA and California.^18^ Aquifers replenish so slowly that they are effectively a non-renewable resource. The depletion of these large freshwater stocks threatens food production and security locally and globally via international food trade. Non-sustainable groundwater abstraction contributed to 20% of global gross irrigation water demand in the year 2000 with this demand having tripled over the period 1960–2000.^19^ For many countries, irrigation is sustained by non-renewable groundwater, and it has been highlighted that ‘a vast majority of the world’s population lives in countries sourcing nearly all their staple crop imports from partners who deplete groundwater to produce these crops’.^18^ Countries who both produce and import food irrigated from rapidly depleting aquifers are particularly at risk, such as USA, Mexico, Iran and China. It has been estimated that India, soon to be the most populous country in the world, will be unable to meet water requirements within 300 years and emerging pressures may reduce this time horizon considerably.^20^ Given the interaction of water supply with energy, this situation may become even worse. For example, in California, 20% of electricity production is used for moving and pumping water for agriculture,^21^ and as water becomes more difficult to access, the energy demand will increase. From a water management point-of-view, the food system can be regarded as unsustainable (cannot meet the ‘continued satisfaction’ requirement) due to its reliance on non-renewable water resources.

Over 20 years ago, it was estimated that around one third of the world’s agricultural land had been lost to erosion and the rate of loss was about 10 Mha/year^22^ Calculations suggest that soil erosion rates under ploughed cultivation are one to two orders of magnitude greater than soil production rates.^23^ This rate of soil loss is not compatible with the ‘continued satisfaction’ requirement for a sustainable food system. It is also linked with other environmental impacts, such as loss of carbon, gaseous emissions, non-point source pollution and sedimentation of waterways,^24^ therefore it is not compatible with the ‘environmentally non-degrading’ criteria as well. Given projections for expansion of dryland areas to around 50% of total land surface, with 78% of dryland expansion in areas supporting 50% of population growth in the coming decades,^25^ the control of soil erosion and its related impacts is going to be a major requirement for sustainable food systems. From a soil management perspective, the food system can be regarded as unsustainable (cannot meet the ‘continued satisfaction’ requirement).

Having considered the energy, nutrient, water, soil and land requirements for food production, it must be concluded that the food system is unsustainable and needs to change because the natural resource base, future satisfaction and environmentally non-degrading requirements cannot be met. It is reasonable to describe food as ‘fossil food’ because of the reliance of non-renewable (and rapidly depleting) resources to supply much of the world’s population. A complete transformation of the food system is required, one that can perhaps be best driven by harnessing appropriate technology to monitor, control and regulate the different types of food system by unleashing the potential benefits of being able to compute over the vast amounts of data that can be obtained from the activities along the food value chain.

### Environmental impact

Modern industrial agriculture was made possible through land clearing and habitat disruption. Some recognised consequences of this were fragmentation and loss of biodiversity, significant greenhouse gas (GHG) emissions from land clearing and adverse impact on marine and freshwater ecosystems.^26^ An estimate suggests that the global food system, from fertiliser manufacture to food storage and packaging, is responsible for up to one third of all human-caused GHG emissions.^27^ Using data from 2005, 2007 and 2008, agricultural production is also estimated to be responsible for a significant share of GHG emissions from the food system, releasing ~12,000 Mt CO_2_e/year representing about 86% of all food-related anthropogenic GHG emissions, followed by fertiliser manufacture at ~575 Mt CO_2_e/year and refrigeration at ~490 Mt CO_2_e/year.^28^ The impacts of such emissions are already being felt^29^ including negative feedbacks on crop yield and health. Reducing this impact will be critical to transitioning from unsustainable fossil food to a sustainable future-proof food system.^28,30^

The eutrophication of surface waters has become an endemic global problem.^31^ From the 1950s to the 1990s, agriculture was associated with a 6.87-fold increase in nitrogen fertilisation, a 3.48-fold increase in phosphorus fertilisation, a 1.68-fold increase in the amount of irrigated cropland and a 1.1-fold increase in land in cultivation.^26^ Agricultural production has been identified as a major underlying and persistent cause of eutrophication in many catchments around the world^32,33^ Nutrient loadings from agriculture are a major driver of water quality deterioration, but it is unclear what level of on-farm control is necessary to achieve water quality improvements.^31^ Smart agriculture and precision farming will drive improvement by increasing resource use efficiency and by harnessing technology to determine current conditions, future weather conditions and the correct intervention.^34,35^

Similar cases can be made for acidification,^36^ biodiversity,^37^ ecosystem toxicity^38^ and other environmental impacts.^39^ Taking just the limited number of examples presented above, it is clear that the ‘environmentally non-degrading’ requirement for a sustainable food system is not being met by current food supply systems and a radical change is needed. From an environmental perspective (resource depletion and adverse impact), it can be concluded that food systems are not sustainable (in general), and if we work from a strong sustainability perspective of working within planetary boundaries,^40^ they cannot become sustainable until this adverse situation is rectified.

## Life cycle thinking methods and the need for an Internet of Food

Life cycle thinking is increasingly being used to assess food system sustainability.^41^ It is an approach used to assess products, processes or services in terms of their place in the world, the full life cycle that is required for them to serve human society and environmental, social and economic consequences of that service. The method has been recognised as the leading approach for including sustainability in decision-making in the United States of America,^42^ Europe^43^ and elsewhere in the world. The quantitative tool used to implement life cycle thinking is life cycle assessment (LCA), which is formalised by international standard (ISO 14040/14044)^44^ and has been widely used to assess food production systems.^45^ LCA is one of the most important methodologies used to assess the impact (pollution and resource depletion) of the food system by using mass and energy flow accounting to model the system and agreed scientific models to calculate resource depletion and specific types of environmental impacts.

It has been suggested that LCA can lead to practitioners focus on the ‘eco-efficiency’ of inherently unsustainable products, and this can lead to increased consumption, because of the LCA paradigm of reducing negatives rather than increasing positives^46^ The cradle-to-cradle (C2C) concept tends to focus more on linking resource consumption and waste creation with sustainability status rather than minimisation of specific impacts. One conclusion is that the best attributes of both approaches should be harnessed.^46^ All such methods (e.g. LCA, C2C) depend on being able to collect sufficient data to characterise a system of interest or the use of publicly funded or commercial databases when site-specific data are not available. It was noted that ‘the practicality of adopting LCA to support decision-making can be limited by the generic nature of the assessment and the resource-intensive nature of data collection and life cycle inventory modelling’,^47^ which is the key limitation for developing tools to facilitate the transition from fossil to sustainable food. The need to share data between stakeholders in increasingly important for the creation of useful information about the food system.

A number of issues associated with using LCA to better understand and manage food systems have been noted,^41^ including (i) the variability of food production, supply chain and consumption globally; (ii) uncertainty related to the specification of data^48^ and the system;^49^ (iii) identifying the boundary between technosphere and ecosphere because agriculture relies on exploiting the ecosphere;^50^ (iv) correctly identifying the real function^51^ of the food system in order to select the most useful functional unit; (v) the multi-functionality of the system; (vi) capturing or modelling inventory data (which requires cooperation between stakeholders for food system applications); (vii) the geo-temporal specificity of background data from LCA databases; (viii) capturing the role of different stakeholders (e.g. consumers, government, industry); (ix) the role of diet choices and (x) handling ‘waste’. These issues are seen in the lack of comparability of LCA studies of the same type of product.^52^ Furthermore, the scope of LCA as a global tool to quantify environmental impacts over the whole life cycle creates limitations.^53^ LCA by its nature, focusses on the global scale and on steady-state, linear homogenous modelling, making it ‘very difficult to include varying spatial and temporal characteristics and nonlinear characteristics of large numbers of processes that occur all over the world’.^53^ There are inherent limitations of inventory because of loss of spatial, temporal, dose–response and threshold information, which reduced the accuracy of impact assessments.^54^ The ‘Internet of Food’ would transform our understanding of the food system and how they are modelled using LCA, provided data sharing is possible. Of the issues affecting food LCA,^41^ most could be directly addressed by the ability to collect data and compute across the whole food chain: variability, uncertainty, multi-functionality, inventory data, databases, stakeholder influence, diet and waste, and the other two, boundary and function, could probably be better understood based on discernible activity. The examples of data mining of U.S. Environmental Protection Agency (EPA) data sets,^47^ potential for avoiding excessive simplification^55^ and use of big data in industrial ecology^56^ indicate that this is the way forward.

## Internet of Food: an enabling technology for the transition from fossil to sustainable

The deployment of sensor networks in the food system have historically been stage-specific and typically designed for monitoring and decision-making at a specific site and time, despite the potential for system integration having been recognised more than a decade ago.^57^ Many sensors have been developed that could be used for the food chain, for example, for soil monitoring, for precision agriculture purposes,^58^ for post-harvest storage monitoring,^59^ for process control,^60^ for retail logistics monitoring^61^ and in some cases for domestic use.^62^ A key requirement to create an ‘Internet of Food’ will be to make the data from these sensors interoperable and to be able to compute across the data set they create. A notable limitation is lack of integration caused by the current mix of open and closed data, communications, hardware standards and a lack of willingness to share data between stakeholders. It has been noted that an ‘…ontology-driven architecture for developing hybrid systems [that] consists of various entities including software components, hardware components (sensors, actuators and controllers), datastores (knowledge base, raw data, metadata), biological elements (plants[or animals]) and environmental context…’^63^ would permit the development of precision agriculture applications, and by logical extension this is required to utilise information across the whole food system (i.e. the Internet of Food). The proposal here is that the ‘hybrid-system’ needs to be extended to cover the whole food system, thus permitting production, process, logistics, retail, purchasing, consumption, nutrition and health outcomes to be integrated through information and computation. Where it is not possible to integrate data of the whole system that delivers a product, it will be very difficult to use Internet of Food for best advantage because its strength is determined by the data available.

A critical requirement will be the development of related ontologies. An ontology is the formal naming of concepts (e.g. types, properties, inter-relationships) within a domain and it is used to describe or infer properties of that domain. In order to be able to draw upon a range of data sources (sensors) and databases (knowledge silos), it is necessary to label data with unique identifiers that permit computers to reason with or compute over those data sets. This is where the real value of Internet of Things, and more specifically Internet of Food lies. To achieve the paradigm shift from fossil food to sustainable food systems, such a shift is needed, facilitated by the ability to reason with such data. As noted,^63^ an ontology-driven architecture is needed to enable the ‘Internet of Food’. Ecologists have recognised the importance of big data in ecological research^64^ in order to address major scientific and societal issues, and to answer the major question facing food (how to achieve a sustainable food system?), an agreed vocabulary and language structure (ontology) is needed. To take simple examples, the word ‘buttermilk’ originally referred to liquid left after churning butter is now also used to describe a fermented or cultured milk drink, so until the language describing these two concepts is standardised it is not possible to compute from diverse data sets within the domain of dairy processing, never mind across domains, where words such as slurry, matrix and texture all mean very different things depending on context. A noted rapid growth of Internet of Things requires standardisation to lower the entry barriers for the new services, to improve interoperability of systems and to allow better services performance.^65^ They noted that this is particularly important for security, communication and identification where interoperability, and particularly semantic interoperability, will be critical. It has been recognised that a proliferation of ad hoc coded data systems will be an impediment to developing data-centric systems that can transform farming,^66^ so sharing of data, agreement of standards and stakeholder cooperation will be required to achieve food systems transformation.

Food ontologies can be used with the specific aim of identifying gaps and for purposes beyond the initial, relatively simple applications, such as recognising foods,^67^ with a contextual focus on diet, food selection, health and wellbeing being possible,^68^ which is a critical component of a sustainable food system, and just as important are the social, economic and environmental impacts and benefits. There are untold opportunities to develop specific services targeted in these areas as well as the potential for integration, with tools such as life cycle sustainability assessment to evaluate the true sustainability of specific food products, meal combinations, whole diets and food systems. These ideas have been evaluated in the context of mining U.S. EPA data for assessing chemical manufacturing,^47^ which identified that automating data access was a challenge because the data are incompatible with semantic queries. Data need to be described using ontologies to relate those data that need to be linked and to introduce LCA concepts to the descriptions. A framework for integrating ‘big data’ with LCA has been suggested^69^ and it was also noted that development of semantic web standards for ecological data have greatly enhanced interoperability in that domain.^70^ The same is required for the food system. It has been suggested that when food (and water) domain descriptors have been developed, this will enable ‘IT support [for] improved production, distribution and sales of foodstuffs [and water]’,^71^ but the development of the domain models for the food chain is perhaps not a task for commerce or industry, rather for public, international research.

The opportunities that will be created by the Internet of Food are immense. One important shift will be from a descriptive, inferential approach to analysing food systems to a ‘big data’ approach.^68^ ‘Big data’ can be characterised in terms of volume (data sets too large for conventional database management), velocity (acquiring, understanding and interpreting data in real time) and variety (the vast array of sources and types of data beyond the conventional rows and columns of numbers describing transactions).^72^ Examples have already emerged where ‘big data’ has been used to provide data useful for LCA including agricultural resource survey^73^ and resource use and emissions associated with U.S. electric power generation.^74^ It is worth pointing out that much of the data relied on for LCA studies is drawn from commonly used databases (e.g. EcoInvent, ELCD, NREL) and are reliant on ‘small data’ and limited observations, which has resulted in reported error (multiple orders of magnitude),^47^ while ‘big data’ offers a means to answering questions about environmental impact or food safety that simply cannot be contemplated in the context of controlled experiments.^71^

Authors have considered ‘big data’ and ‘internet of things’ in the context of specific parts of the food chain. For example, ‘big data’ in ‘smart farming’ (i.e. the production stage of the food system) is now being used to make predictive insights about farm operations, to support operational decisions, to redesign business processes and to change business models.^75^ To leverage this value at the farm level required extension along the food chain beyond the farm, but two scenarios are emerging: closed proprietary systems and open collaborative systems,^74^ such as Food Industry Intelligence Network,^76^ Food Innovation Network^77^ and European Institute of Innovation & Technology (EIT) Food.^78^ Priority should be given to development of data and applications infrastructure and at the same time to organisational issues concerning governance and business models for data sharing.^74^ In the context of circular economy (i.e. the end of life, non-consumed part of the food system), it was found that, despite the concept of circular economy being discussed for decades, it has not become an adopted business model.^79^ An analysis of literature from 2006 to 2015 found only 70 publications at the intersection of circular economy and ‘big data’/‘internet of things’, but nearly half (34) had been published in 2015.^75^ It was suggested that technology encompassed by ‘big data’ and ‘internet of things’ is what is needed to enable such change,^75^ which is the same argument being put forward here for the Internet of Food in the context of sustainable food systems. Two implications of relevance for the Internet of Food are: there is a gap between scientific research and corporate initiatives, which needs to be closed, and the search of literature was limited by the keywords available, and more specifically the lack of structured taxonomy to describe the circular economy. It is reasonable to conclude that if these ideas are relevant to one small component of the Internet of Food, then they are probably relevant to the concept as a whole.

These two recent reviews highlight the importance of developing the Internet of Food as a precompetitive platform on which business models can be built, much like the internet as we currently know it, and to achieve this we need to define agreed vocabularies and ontologies to be able to reason and compute across the vast amounts of data that are and will be available in the future. The ability to compute over large amounts of data will change the way the food system is analysed and understood. Biological scientists have noted how important data curation is, because as curated data become available the way science is conducted changes.^80^ A key requirement of data curation is the connection of data from different sources in a human- and machine-comprehensible way. Another key change is the processing of multiple sources of complex data (‘big data’) using inference programs. While this might lead to new ways of conducting experimental (hypothesis driven) research, it is also unlocking the door to data-driven research, i.e. extracting new knowledge and understanding from data without experimentation or preconceived ideas, and providing new management approaches based on information and better decision-making capabilities.^71^

The Internet of Food offers substantial opportunities for understanding the limits and constraints to sustainable food systems and thus supporting decisions about the transition from fossil to sustainable food. It is essential that all stakeholders engage with the development of Internet of Food to ensure harmonious development of a technology that can be used for both pre-competitive applications and commercial exploitation, if it is to be fully developed over the coming 5–10 years. In addition to the technical issues highlighted here, there are considerations of data ownership, privacy, ethical use of data, market control and other application domains (e.g. food safety, traceability, personal nutrition, security, fraud and policy) that need to be developed with stakeholder contributions alongside the technical advances considered here.

## Conclusions

In order to transition to a sustainable food system, we need specific technology infrastructure to allow high-quality data to be collected about the food system that will permit the best possible decision-making. Key requirements are: standard vocabularies and ontologies to allow integration of data sets across the internet; proliferation of low cost sensing to allow orders of magnitude change in the supply of empirical observation data into LCA models; and new analytical methods to collate, curate, analyse and utilise data across the whole food production system. We need an Internet of Food to monitor conditions and analyse data to derive knowledge that can be combined with the means to implement control of the system to enable a step change in how we think about food systems. This technology will give us the chance to transition from fossil food to sustainable food systems.
